# Neuron-to-vessel signaling is a required feature of aberrant stem cell commitment after soft tissue trauma

**DOI:** 10.1038/s41413-022-00216-x

**Published:** 2022-06-01

**Authors:** Qizhi Qin, Mario Gomez-Salazar, Masnsen Cherief, Chase A. Pagani, Seungyong Lee, Charles Hwang, Robert J. Tower, Sharon Onggo, Yuxiao Sun, Abhinav Piplani, Zhao Li, Sowmya Ramesh, Thomas L. Clemens, Benjamin Levi, Aaron W. James

**Affiliations:** 1 Department of Pathology, Johns Hopkins University, Baltimore, MD 21205 USA; 2 Center for Organogenesis and Trauma, Department of Surgery, University of Texas, Southwestern, TX USA; 3 Department of Plastic Surgery, Harvard, Cambridge, MA 02138 USA; 4 Department of Orthopaedics, Johns Hopkins University, Baltimore, MD 21205 USA; 5 Baltimore Veterans Administration Medical Center, Baltimore, MD 21201 USA

**Keywords:** Bone, Bone quality and biomechanics

## Abstract

The functional interdependence of nerves and blood vessels is a well-established concept during tissue morphogenesis, yet the role of neurovascular coupling in proper and aberrant tissue repair is an emerging field of interest. Here, we sought to define the regulatory relationship of peripheral nerves on vasculature in a severe extremity trauma model in mice, which results in aberrant cell fate and heterotopic ossification (HO). First, a high spatial degree of neurovascular congruency was observed to exist within extremity injury associated heterotopic ossification. Vascular and perivascular cells demonstrate characteristic responses to injury, as assessed by single cell RNA sequencing. This vascular response to injury was blunted in neurectomized mice, including a decrease in endothelial proliferation and type H vessel formation, and a downregulation of key transcriptional networks associated with angiogenesis. Independent mechanisms to chemically or genetically inhibit axonal ingrowth led to similar deficits in HO site angiogenesis, a reduction in type H vessels, and heterotopic bone formation. Finally, a combination of single cell transcriptomic approaches within the dorsal root ganglia identified key neural-derived angiogenic paracrine factors that may mediate neuron-to-vascular signaling in HO. These data provide further understanding of nerve-to-vessel crosstalk in traumatized soft tissues, which may reflect a key determinant of mesenchymal progenitor cell fate after injury.

## Introduction

A high degree of neurovascular congruency exists within development, in which peripheral nerves and blood vessels grow in tandem toward target tissues.^1,2^ The intricate co-dependency of peripheral nerves and vasculature is intuitive, as blood vessels transfer nutrients to axons, while peripheral nerves control vessel caliber.^3^ Re-growth of neurovasculature into injured tissues is also tightly coordinated, and in particular in mammalian skeletal repair our group and others have shown that vascular assembly is critically dependent on proper tissue re-innervation.^4–6^ Here, inhibition of Nerve growth factor (NGF) responsive TrkA (tropomyosin receptor kinase A)-expressing skeletal sensory nerves led to secondary deleterious effects on injury site vascularity.^4,5^ This is in line with literature in other organ systems, in which for example nerves influence the trajectory of blood vessels and specification of the arterial system during embryogenesis within skin.^7,8^ Such observations of neurovascular coupling within the skeleton were suggested in elegant anatomic studies of the developing avian limb.^9–11^ Yet, neuron-to-vessel signaling, its importance and molecular mediators in musculoskeletal trauma, is a vastly understudied subject.

Severe reactions in soft tissue trauma can result in aberrant osteochondral differentiation of resident mesenchymal progenitor cells, in a process collectively termed heterotopic ossification (HO).^12–15^ In its more severe manifestations, HO can induce chronic pain, impair wound healing, increase medical care utilization, lead to disability, and reduce quality of life.^16,17^ Pain precedes HO in sporadic forms,^18–20^ and even rare genetic forms,^21–23^ which is often out of proportion to clinical examination. Experimental animal models of HO recently reported by our group observed that tendon-associated HO is dependent on the presence and/or activity of TrkA-expressing sensory neurons^6^—findings in agreement with other observations of skeletal ossification.^24,25^ In fact, a large body of literature has demonstrated the importance of crosstalk between neural and skeletal systems during development, suggesting a neuro-osteogenic network.^26^ Within the tendon-associated HO model, chemical or surgical means to block injury site re-innervation led to partial or complete reductions in HO formation.^6^ It appears that BMP2 induced HO has a similar neural requirement,^24,27^ although whether this extends to genetic forms of HO is not clear. In addition, osteogenesis and angiogenesis have been studied as coupled processes during skeletal morphogenesis.^28^ A similar phenomenon is apparent in HO, where independently our group identified a role for vascular endothelial growth factor A (VEGFA) signaling^29^ and hypoxia^30^ in driving a supportive angiogenic niche for HO formation. Despite these independent observations, the potential for neuron-to-vascular signaling in the regulation of heterotopic bone has not been explored.

Here, we sought to define the regulatory relationship of peripheral nerve signaling on the vasculature in a previously characterized trauma-induced HO model of aberrant cell fate.^31–33^ Overall, a high degree of spatial neurovascular congruency was observed in trauma-associated HO. Single cell RNA sequencing (scRNA-Seq) identified that neurectomized mice demonstrated shifts in endothelial cell phenotype which precede the development of HO. Independent mechanisms to chemically inhibit axonal ingrowth led to deficits in HO site angiogenesis and a reduction in type H vessel formation. Finally, a combination of transcriptomic approaches identified key neural-derived angiogenic paracrine factors that may mediate neuron-to-vascular signaling in HO.

## Results

### Neurovascular ingrowth after soft tissue trauma accompanies transcriptional changes within vascular cells

**Fig. 1:**
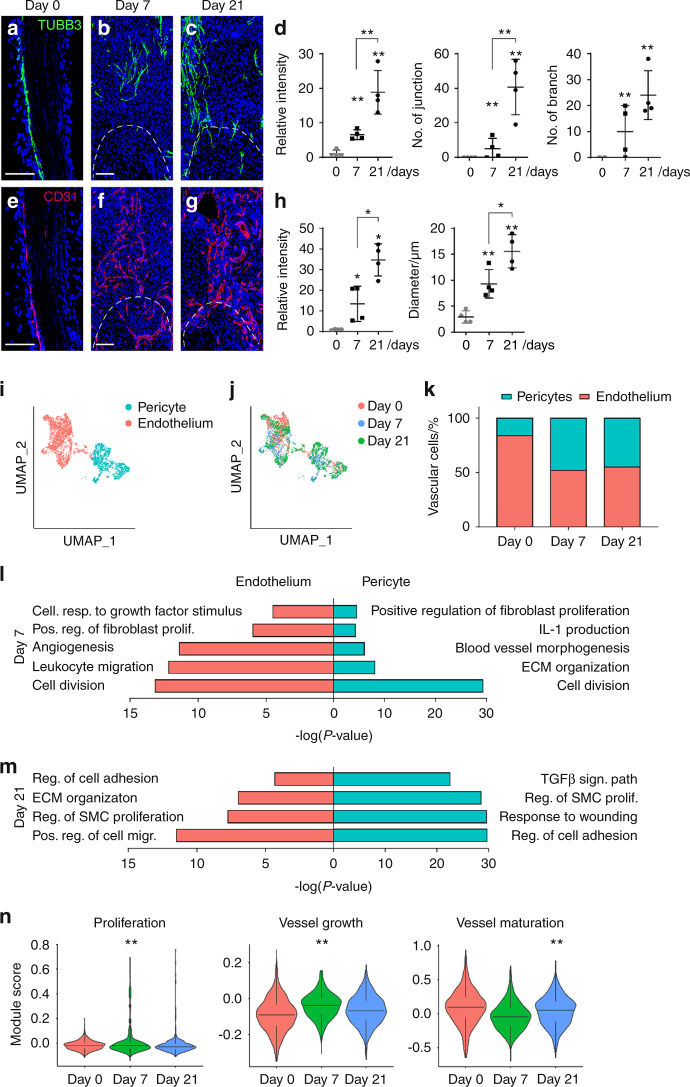
Neurovascular ingrowth and transcriptional profile after soft tissue trauma. Traumatic injury (complete Achilles tenotomy with dorsal burn to induce heterotopic ossification) was performed on 6–8 weeks old, male and female C57BL/6 J mice, with analyses at 7 and 21 days. Day 0 indicates uninjured control. **a**–**c** Representative pan-neuronal Beta III Tubulin (TUBB3) immunofluorescent staining within sagittal sections of tendon injury site, day 0, 7 and 21 post-tenotomy. **d** Quantification of TUBB3^+^ nerves within the injury site over time, including overall TUBB3 staining intensity, number of nerve junctions, and number of nerve branches. **e**–**g** Representative immunofluorescent staining of the endothelial marker CD31 over the 21-day time course post-injury. **h** Quantification of CD31^+^ blood vessels within the injury site over time, including overall CD31 staining intensity and vessel diameter. **i**–**m** Single cell RNA sequencing analysis of pooled cells from the injury site, obtained at day 0, 7 and 21 post-injury. **i** UMAP visualization of endothelial and pericyte clusters at all timepoints, and (**j**) UMAP visualization indicating cells at day 0, 7 and 21 post-injury. **k** Percentage of endothelial cells and pericytes across time as a function of both cell types combined. **l, m** GO term analysis of endothelial cells (red) and pericytes (blue) at day 7 and day 21 post injury in relation to uninjured control. **n** Modular index scoring of cell proliferation, vessel growth, and vessel maturation in endothelial cells across time points. Gene lists for modular scores are shown in File S1. Dashed white lines indicate edges of Achilles tendon. Scale bars: 100 μm. For histology, *n* = 4 animals per timepoint. For scRNA-Seq, *n* = 3 animals per timepoint. Individual dots in scatterplots represent values from single animal measurements, while mean and one SD are indicated by crosshairs and whiskers. *P < 0.05; **P < 0.01 in relation to D 0 timepoint. Two-tailed student’s *t* test was used for two-group comparisons (**b**, **d**). For more than two groups, one-way ANOVA followed by post hoc analysis was performed (**n**). Non-parametric data was analyzed using the Kruskal–wallis with Dunn’s multiple comparison test (**n**)

In prior work, we observed neuronal sprouting to occur during the development of extremity trauma associated heterotopic bone.^6^ Using the same model of HO induction, we sought to confirm this finding and relate axonal ingrowth to vascular proliferation and maturation (Fig. 1a–d, Fig. S1). Confirming prior findings,^6^ innervation of the uninjured tendon was limited and confined to the peritenon as demonstrated by Beta III Tubulin immunofluorescent staining (TUBB3, Fig. 1a, left). Upon HO inducing tenotomy, brisk ingrowth of tendon-associated nerves was found at day 7 (previously characterized fibroproliferative phase of injury,^6^ Fig. 1b) and progressed at day 21 (previously characterized cartilaginous phase of injury,^6^ (Fig. 1c). Quantification of TUBB3^+^ axons confirmed an increase in overall staining intensity, but also an increasing complexity of neuronal projections with increasing junctions and branchpoints overtime (Fig. 1d). CD31 immunofluorescent staining was performed to highlight angiogenesis over the same time course (Fig. 1e–g). A similar spatial pattern of vascular growth was observed, in which endothelium was principally confined to the peritenon among uninjured tendons (Fig. 1e), and a progressive increase in CD31^+^ vascular channels was found at 7 and 21 days after HO induction (Fig. 1f, g). In parallel to changes in neuronal histomorphometry, quantification of vascularity demonstrated similar kinetics of progressive increase in overall CD31^+^ endothelium, as well as an increase in the mean diameter of vascular channels, presumed to reflect a more mature vascular network (Fig. 1h).

Single cell RNA Sequencing (scRNA-Seq) profiling of the HO induction site was next examined within this time frame (Fig. 1i–m). Of 22,902 total cells, 4043 cells were identified as endothelial cells (*Pecam1*^high^, *Cdh5*^high^) or pericytes (*Acta2*^high^, *Pdgfrb*^high^) for further analysis (Fig. 1i, Fig. S2). Both cell clusters were represented across all timepoints (Fig. 1j), with a relative enrichment in pericyte frequency after injury (Fig. 1k). To characterize the vascular response to traumatic injury, GO term analysis of both endothelial cells and pericytes was performed at 7 and 21 days in comparison to uninjured control (Fig. 1l, m). Seven days after injury, both vascular cell types showed enrichment in GO terms related to cell proliferation, angiogenesis, and the regulation of inflammation (Fig. 1l). After 21 days, vascular cell types showed enrichment for GO terms related to smooth muscle cell proliferation, cell adhesion and migration, matrix organization, and TGFβ signaling (Fig. 1m). Having observed these dynamic changes over time, we next examined shifts in endothelial cells using gene modules related to cell proliferation, vessel growth and maturation (Fig. 1n). Confirming prior findings, module scorings of proliferation and vessel growth acutely increased at 1 week post-injury, and after 3 weeks re-approximated the state of uninjured endothelium. In contrast, a module score of ‘vessel maturation’ demonstrated the inverse trend, in which an acute reduction was observed at 1 week post-injury, which re-approximated the score of uninjured endothelium at 3 weeks post-injury. Thus, neurovascular growth and network maturation accompanies trauma-induced HO, and the period between 1 and 3 weeks after injury is a dynamic time frame to examine this process on a transcriptional and histologic level.

### Endothelial cell subclustering highlights an increase in type H vessels after HO induction

**Fig. 2:**
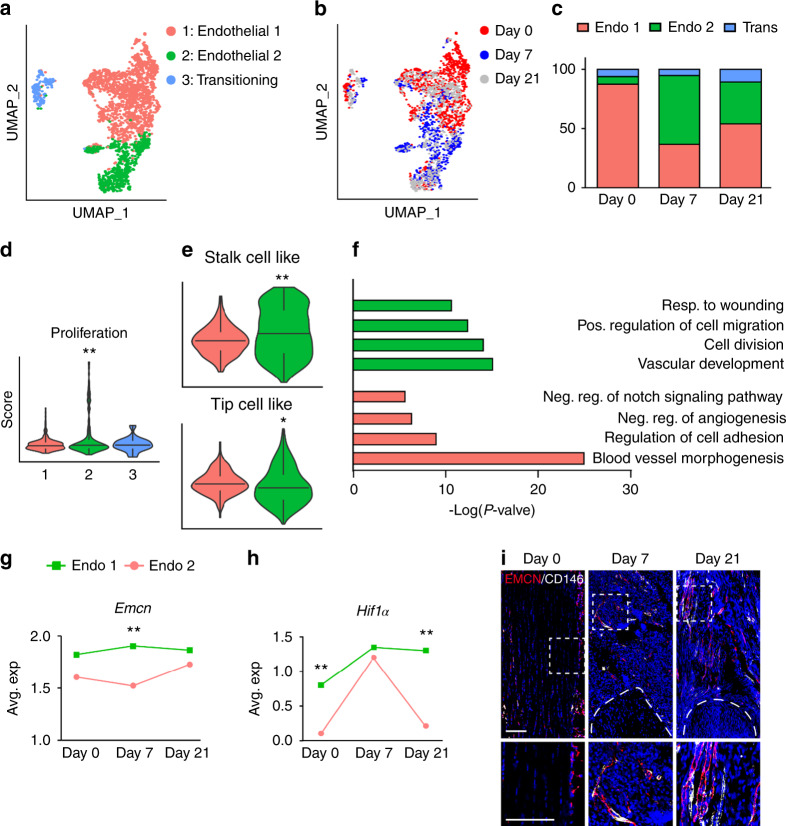
Endothelial cell subclustering reveals distinct cell populations after soft tissue trauma. **a**–**h** ScRNA-seq analysis of pooled endothelial cells from the HO induction site at 0, 7 and 21 days post-injury. **a, b** UMAP visualization of all endothelial cells demonstrating (**a**) endothelial subclusters, and (**b**) cells across time (day 0, day 7, and day 21). **c** Percentage of endothelial cell subclusters across time. **d** Cell proliferation index across endothelial cell subclusters as determined by modular scoring, day 7. **e** Modular index scoring of stalk cell-like and tip cell-like phenotypes in endothelial subclusters. Gene lists for modular scores are shown in File S1. **f** GO term analysis of endothelial 1 and endothelial 2 subsets, based on differentially expressed genes between the groups. **g** *Emcn* expression among endothelial sub-clusters across time. **h** *Hif1α* (Hypoxia-inducible factor 1-alpha) expression among endothelial sub-clusters across time. **i** Representative immunofluorescent staining of EMCN (Endomucin) and CD146 within the tendon injury site at day 7 and 21 in relation to uninjured control (D0). Dashed white lines indicate edges of Achilles tendon. Scale bars: 100 μm. For histology, *n* = 4 animals per timepoint. For scRNA-Seq, *n* = 3 animals per timepoint. *P < 0.05; **P < 0.01 in relation to endothelial subcluster 1. For two groups, non-parametric data was analyzed using the Kolmogorov–Smirnov test (**e**, **g**, **h**). For more than two groups, Non-parametric data was analyzed using the Kruskal–wallis with Dunn’s multiple comparison test (**d**)

Having demonstrated shifts in endothelial cell phenotype after injury, endothelial cell subclustering was next performed (Fig. 2). Upon re-clustering, three subclusters of *Pecam1*^high^, *Cdh5*^high^ cells were identified: Endothelial 1, endothelial 2, and a small ‘transitioning’ cell population (Fig. 2a), the latter of which expressed a combination of endothelial and pericyte markers (e.g., *Mcam*, *Pdgfrb*, *Acta2*). All populations were represented before and after HO induction, however endothelial 2 was greatly overrepresented at 7 and 21 days in comparison to baseline uninjured samples (Fig. 2b, c, 58.0% and 35.4% of endothelial cells at 7 and 21 days in comparison to 6.4% at baseline). Characterization of the endothelial 2 cell subcluster revealed a high proliferation phenotype, as determined by module score in relation to other cell clusters (Fig. 2d). Tip cells and stalk cells represent two distinct endothelial cell phenotypes in the nascent sprout of vessels, one guides the growing vessel (tip) and the other that proliferates and follows (stalk).^34^ Further transcriptomic characterization revealed a stalk-like cell phenotype in the endothelial 2 subcluster, while endothelial 1 demonstrated enrichment for a tip cell-like phenotype, using module scoring (Fig. 2e). GO term analysis of these clusters again demonstrated relative enrichment in terms related to proliferation, angiogenesis and response to extremity injury among the endothelial 2 subcluster, whereas endothelial cluster 1 shows a regulatory phenotype (Fig. 2f) giving further evidence of tip and stalk cell phenotypes.

Much attention has been paid to endothelial subtypes involved in osteo-angiogenic coupling, including type H vessels characterized by high expression of endomucin (*Emcn*).^35^ To our knowledge, the documentation of type H vessels in HO has not been performed. Indeed, we observed that the endothelial 2 subcluster expressed higher levels of *Emcn* in comparison to the endothelial 1 subcluster (Fig. 2g). Moreover, the endothelial 2 subcluster demonstrated higher expression of *Hif1α* (Hypoxia inducible factor 1 subunit alpha) at baseline, and upon HO induction - high *Hif1α* expression has been previously linked to a type H vessel phenotype^36^ (Fig. 2h). Results thus far by scRNA-Seq suggested early expansion of a proliferative endothelial cell subset with markers suggestive of type H vessel identity. To further examine this, immunofluorescent staining for Emcn was performed of the injury site in relation to uninjured control, using co-immunohistochemistry for the pan-vascular marker CD146 (Fig. 2i). Results showed infrequent Emcn^high^ vessels within uninjured tendon, primarily restricted to the peritenon. A progressive increase in Emcn^high^ vessels was observed at 7 and 21 days after HO inducing injury. Together, these data suggest an increase of endothelial cells with a type H phenotype at early timepoints in the evolution of trauma-induced HO. To our knowledge this is the first time that type H vessels have been reported in the context of HO which contributes to our understanding of this pathology and may help better develop new strategies for treatment.

### Denervation prevents vascular ingrowth and shifts endothelial phenotype after soft tissue trauma

**Fig. 3:**
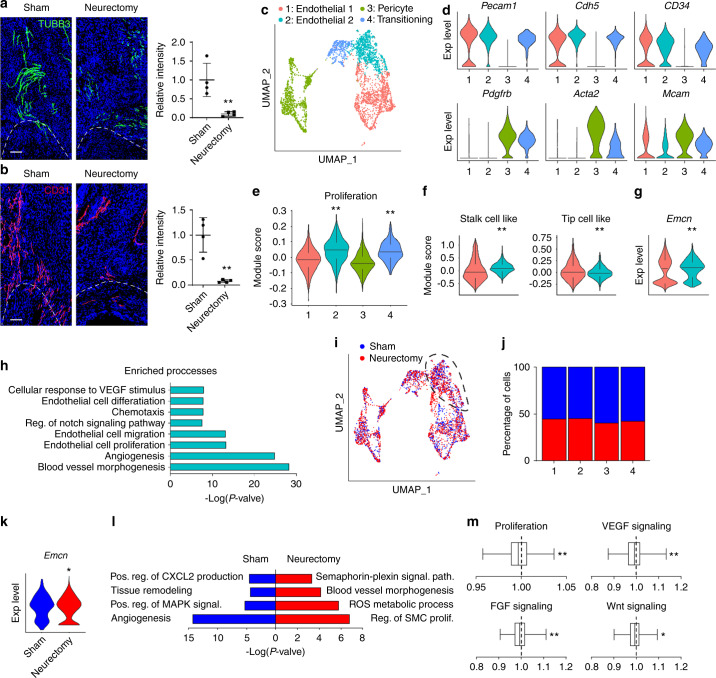
Neurectomy impairs blood vessel infiltration after soft tissue trauma. Animals underwent proximal sciatic neurectomy or sham surgery at the time of HO induction. **a** TUBB3 (Beta III Tubulin) immunostaining and quantification within the tendon injury site among sham or neurectomized mice, 21 d post-injury. **b** CD31 immunostaining and quantification within the tendon injury site among sham or neurectomized mice, 21 d post-injury. Dashed white lines indicate edges of Achilles tendon. **c**–**l** ScRNA-seq analysis of the HO site at 7 d post injury, among sham or neurectomized animals. **c** UMAP visualization of subclustering of endothelial and pericyte populations. **d** Violin plots of characteristic gene markers identifying four vascular subclusters. *Pecam1* (Platelet endothelial cell adhesion molecule 1), *Cdh5* (Cadherin 5), *Pdgfrb* (Platelet-derived growth factor receptor beta), *Acta2* (Actin alpha 2), *Mcam* (Melanoma cell adhesion molecule). **e** Modular index scoring for cell proliferation across subclusters. **f** Module index scoring for ‘stalk cell-like’ and ‘tip cell-like’ phenotypes across Endothelial 1 and Endothelial 2 subclusters. **g** *Emcn* expression across Endothelial 1 and Endothelial 2 subclusters, by violin plot. **h** GO terms enriched in Endothelial 2 subcluster as compared to Endothelial 1. **i** UMAP visualization of vascular cell subclusters with or without sciatic neurectomy. **j** Cellular percentage of Endothelial 2 subcluster with or without sciatic neurectomy. **k** *Emcn* expression in Endothelial 2 subcluster in the context of sham or neurectomy conditions, as shown by violin plot. **l** GO term analysis of endothelial 2 subcluster in the context of sham and neurectomy surgery. **m** Module index scoring of proliferation, VEGF (Vascular endothelial growth factor) signaling, FGF (Fibroblast growth factor) signaling, and Wnt signaling under neurectomy conditions in comparison to sham control. Values greater than 1 indicates increased signaling activation in neurectomy group while values less than 1 indicates reduced expression among the neurectomy group. Scale bars: 100 μm. For histology, *n* = 4 animals per condition. For scRNA-Seq, *n* = 3 animals per condition. Individual dots in scatterplots represent values from single animal measurements, while mean and one SD are indicated by crosshairs and whiskers. *P < 0.05; **P < 0.01 in relation to endothelial subcluster 1 (**e**–**g**) or sham control (**a**, **b**, **k**, **l**). A two-tailed student’s *t* test was used for two-group comparisons (**a**, **b**, **f**, **g**, **k**, **l**). For more than two groups, non-parametric data was analyzed using the Kruskal–wallis with Dunn’s multiple comparison test (**e**). Non-parametric data was analyzed using the Kolmogorov–Smirnov test (**f**, **g**, **m**)

Our previous studies demonstrated that surgical denervation via the use of sciatic neurectomy largely prevented the formation of HO in our tendon injury model (97.0% reduction in bone formation as determined by microCT imaging analysis).^6^ However, the extent to which vascularity was altered with surgical denervation (i.e., the dependence of an HO-associated angiogenic response on intact innervation) had not been assessed. Here, ipsilateral sciatic neurectomy (or sham surgery with visualization only of the nerve) was performed at the time of HO induction (Fig. 3). As expected and confirming prior results,^6^ sciatic neurectomy led to a 90.1% reduction in nerve fiber frequency at the HO site (Fig. 3a, 21 d post-injury, assessed by TUBB3 immunofluorescent staining). Next, CD31 immunofluorescent staining showed a similarly dramatic reduction in vascularity after sciatic neurectomy (Fig. 3b, 92.4% reduction in CD31 immunostaining in comparison to sham control).

To further understand the transcriptomic changes in vascular cells with or without intact innervation, re-analysis of our prior scRNA-Seq data was performed in which HO induction was performed combined with or without sciatic neurectomy, and cells of the HO site were analyzed at 7 days after injury (GSE163446).^6^ Of a total of 16 823 cells, 3 616 vascular cells were captured and distributed across 4 subclusters with strong similarity to the previously analyzed dataset, including endothelial cell 1, endothelial 2, pericytes, and again a ‘transitioning’ cluster with markers shared between endothelial and pericytes (Fig. 3c, d). Consistent with prior observations, the endothelial 2 subcluster showed the highest proliferative index by module scoring (Fig. 3e), expression more consistent with a stalk cell-like phenotype (Fig. 3f), and high *Emcn* expression (Fig. 3g). GO term analysis for the endothelial 2 subcluster again showed relative enrichment for biological processes such as endothelial proliferation, migration and angiogenesis (Fig. 3h). Thus, this scRNA-Seq dataset demonstrated overall similar vascular cell populations for analysis, and endothelial subcluster 2 again appeared to represent a proliferative, type H-like endothelium appropriate for focused comparison.

Next, comparisons were performed between sham and neurectomy conditions (Fig. 3i–m). Each vascular subcluster was present under both sham and neurectomy conditions (Fig. 3i), and showed overall a slight increase in frequency across sham as compared to neurectomy samples (Fig. 3j). The endothelial 2 subcluster showed a shift in phenotype in response to neurectomy as observed by a reduction in *Emcn* expression (Fig. 3k), as well as GO term analysis (Fig. 3l). For example, terms related to angiogenesis, tissue remodeling and MAPK signaling were enriched among control (sham operated) conditions. Focused analysis of specific pathways was further undertaken using gene module scores (Fig. 3m). Proliferative indices were reduced under neurectomy conditions. Signaling pathway changes were also observed including reductions in VEGF, FGF, and WNT signaling pathway scoring among endothelial cells of neurectomized mice (Fig. 3m). Thus, surgical denervation blunts HO-associated angiogenesis accompanied by a shift in endothelial cell transcriptional phenotypes, which may be requisite features of HO formation and provides new insights into molecular pathways that could be targeted.

### Pharmaco-genetic or pharmacological inhibition of TrkA signaling impairs angiogenesis at sites of trauma

**Fig. 4:**
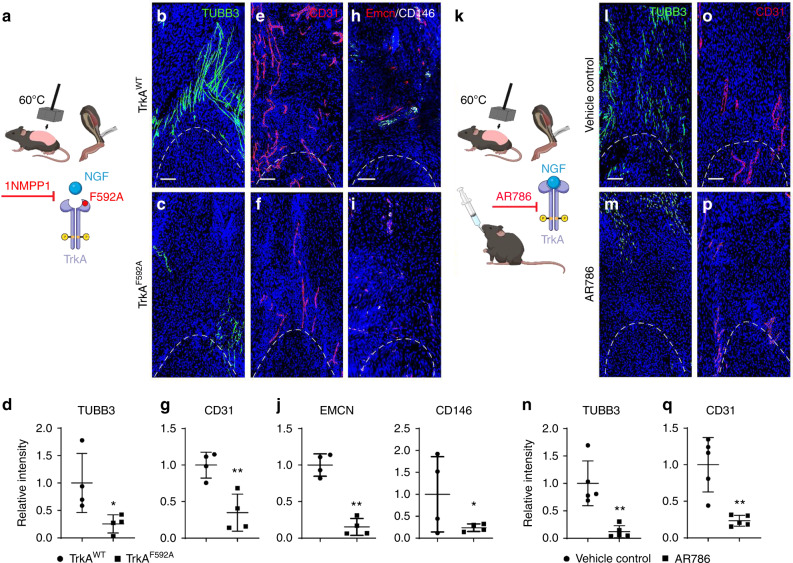
Genetic and pharmacological inhibition of TrkA signaling impairs vascular ingrowth after soft tissue trauma. **a** Schematic representation of experiment. (Created in Biorender.com). TrkA^F592A^ mice have a point mutation which renders them susceptible to temporally controllable inhibition of TrkA catalytic activity, via administration of the small molecule 1NMPP1. Either TrkA^WT^ or TrkA^F592A^ animals (6–8 weeks old, male and female mice) were subjected to HO induction and both administered 1NMPP1 throughout the study period. Analyses performed at 21 d post-injury. **b**–**d** Representative immunohistochemical staining for TUBB3 (Beta III Tubulin, appearing green) and quantification among TrkA^WT^ or TrkA^F592A^ animals, as assessed at the distal tenotomy site as visualized using sagittal cross-sections. **e**–**g** Representative immunostaining for CD31 (appearing red) and quantification among TrkA^WT^ or TrkA^F592A^ animals. **h**–**j** EMCN (Endomucin) and CD146 co-immunohistochemical staining (appearing red and white) and quantification among TrkA^WT^ or TrkA^F592A^ animals. **k** Schematic representation of experiment. C57BL/6 J mice (6–8 weeks old, male and female mice) were administered AR786 or vehicle control throughout the study period. HO induction was performed (burn/tenotomy), with analysis at day 21. **l**–**n** Representative immunohistochemical staining for TUBB3 and quantification among control or AR786 treated animals, as assessed at the distal tenotomy site. **o**–**q** Representative immunostaining for CD31 (appearing red) and quantification among control or AR786 treated animals. Dashed white lines indicate edges of Achilles tendon. Scale bars: 100 μm. *n* = 4–5 animals per group. Individual dots in scatterplots represent values from single animal measurements, while mean and one SD are indicated by crosshairs and whiskers. * *P* < 0.05; ** *P* < 0.01. A two-tailed student’s *t* test was used for all comparisons

Surgical denervation using the sciatic nerve represents a mixed motor-sensory deficit which may have multiple parallel effects on angiogenesis and HO formation within the extremity. Next, two established models were assessed to specifically inhibit TrkA-expressing nerves within the context of an HO inducing injury (Fig. 4). Here, TrkA-expressing nerve ingrowth was first inhibited using a chemical-genetic approach, in which a point mutation in TrkA (TrkA^F592A^) renders mutant mice susceptible to small molecule mediated inhibition of TrkA catalytic activity using the small molecule 1NMPP1 (Fig. 4a). Previously, this animal showed 74.1% reduction in heterotopic bone by microCT imaging in comparison to wildtype control animals.^6^ In similarity to past observations,^6^ TrkA^F592A^ mutant animals showed a prominent reduction in HO site innervation (Fig. 4b, c), quantified as a 74.5% reduction (Fig. 4d). Injury site vascularity was next assessed in TrkA^F592A^ mutant animals using CD31 immunofluorescent staining. Results showed a significant impairment in HO site angiogenesis in the context of TrkA inhibition (Fig. 4e, f), quantified as a 65.1% reduction in overall staining intensity (Fig. 4g). Emcn and CD146 co-immunofluorescent staining and quantification was next performed (Fig. 4h–j). Here, a similar reduction in injury site vascularity was observed among TrkA mutant animals, including a notable reduction in the numbers of Emcn^high^ blood vessels (84.8% reduction at 21 d post injury), as well as CD146 (85.9% reduction at 21 d post injury). Next, TrkA inhibition was achieved in a parallel method by oral administration of the small molecule inhibitor AR786 to wildtype mice during the formation HO (Fig. 4k). In our past work, AR786 led to a prominent reduction in the amount of cartilage after HO induction (69.3% reduction at 21 d after injury^6^). As in prior experiments, TrkA inhibition via AR786 treatment led to a significant reduction in injury site innervation (Fig. 4l–n, 88.1% reduction), but also led to a prominent reduction in vascular density (Fig. 4o–q, 76.6% reduction). Thus, three independent methods to inhibit TrkA-expressing sensory nerve ingrowth led to significant deficits in extremity injury associated angiogenesis, further supporting neuron-to-endothelial signaling after musculoskeletal trauma.

### Traumatic HO incites a pro-angiogenic gene profile among sensory neurons

**Fig. 5:**
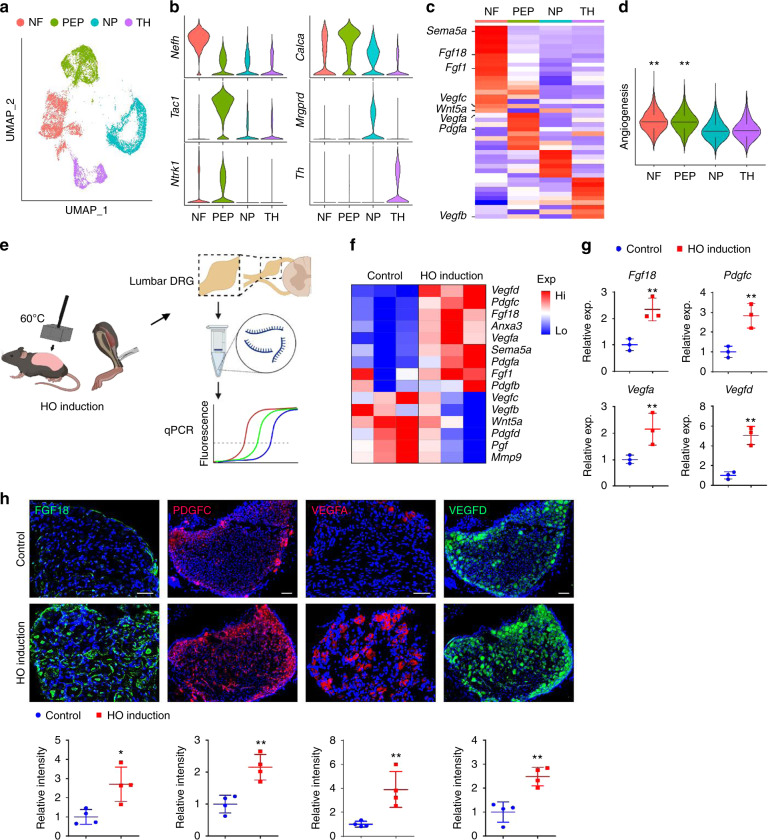
TrkA^+^ peripheral nerves express key angiogenic genes after soft tissue trauma. **a**–**d** Re-analysis of scRNA-Seq of mouse lumbar DRG neurons (8–12 weeks old male C57BL/6 J mice, *n* = 3 mice, 11 191 neuronal DRG cells were examined) (**a**) UMAP visualization of 4 cell clusters including Neurofilament (NF), Peptidergic nociceptors (PEP), Non-peptidergic (NP) and Tyrosine hydroxylase (TH) in pooled cells. **b** Violin plots demonstrating characteristic markers of each cell cluster. *Nefh* (Neurofilament Heavy Chain), *Calca* (Calcitonin Related Polypeptide Alpha), *Tac1* (Tachykinin Precursor 1), *Mrgprd* (MAS Related GPR Family Member D), *Ntrk1* (Neurotrophic Receptor Tyrosine Kinase 1), *Th* (Tyrosine Hydroxylase). **c** Heatmap of angiogenic gene expression among each cell cluster. *Sema5a* (Semaphorin 5 A), *Fgf18* (Fibroblast Growth Factor 18), *Fgf1* (Fibroblast Growth Factor 1), *Vegfc* (Vascular Endothelial Growth Factor C), *Wnt5a* (Wnt Family Member 5 A), *Vegfa* (Vascular Endothelial Growth Factor A), *Pdgfa* (Platelet Derived Growth Factor Subunit A), *Vegfb* (Vascular Endothelial Growth Factor B). **d** Module score analysis of angiogenic genes among each cell cluster. **e**–**g** qPCR analysis of angiogenic gene expression among ipsilateral lumbar DRGs after HO induction, in comparison to uninjured control. Ipsilateral L4-L6 pooled for analysis. **e** Schematic of qPCR analysis of angiogenic gene expression among ipsilateral lumbar DRGs after HO induction. Created in Biorender.com. **f** Heatmap of 15 pro-angiogenic genes enriched in *Ntrk1*-expressing neurons after HO induction, in comparison with uninjured control. Analysis performed at 3 d post-injury. **g** Representative most up-regulated genes upon HO induction, including *Fgf18*, *Pdgfc, Vegfa*, and *Vegfd*. **h** Representative immunofluorescent staining of ipsilateral L4 DRG after HO induction in relation to uninjured control, including FGF18 (Fibroblast Growth Factor 18), PDGFC (Platelet Derived Growth Factor Subunit C), VEGFA (Vascular Endothelial Growth Factor A), and VEGFD (Vascular Endothelial Growth Factor D). Analysis performed at 3 day post-injury, and quantification performed in relation to uninjured control. Scale bars: 100 μm. *n* = 3 or 4 animals per group. Individual dots in scatterplots represent values from single animal measurements, while mean and one SD are indicated by crosshairs and whiskers. * *P* < 0.05; ** *P* < 0.01. A two-tailed student’s *t* test was used (**g**, **h**). Non-parametric data was analyzed using the Kolmogorov–Smirnov test (**d**)

Having shown the dependence of neural ingrowth for angiogenesis at sites of HO predisposing trauma, we next set out to identify potential vasculogenic growth factors enriched within sensory neural populations. First, re-analysis of a scRNA-Seq dataset of mouse dorsal root ganglia (DRG) cells was performed (8–12 weeks old C57BL/6 J mice).^37^ In agreement with prior reports, four main cell clusters were identified, including: Neurofilament (NF), peptidergic nociceptors (PEP), non-peptidergic (NP) and tyrosine hydroxylase (TH) based on the expression of specific markers (Fig. 5a, b). *Ntrk*1 (encoding TrkA) was found in peptidergic cells and to a lesser extent in NF cells (Fig. 5b). Next, candidate secreted pro-vasculogenic factors were examined in this dataset. Heatmap demonstration showed unique angiogenic gene profiles across cell clusters (Fig. 5c). For instance, among the *Ntrk1*^low^ NF neurons *Sema5a*, *Fgf18*, *Fgf1*, *Vegfc* and *Wnt5a* demonstrated enrichment. Among the *Ntrk1*^high^ PEP neurons, *Vegfa* and *Pdgfa* among others showed enrichment. The impression that *Ntrk1*-expressing neurons showed high expression of vasculogenic regulatory genes was confirmed via module score analysis, in which NF and PEP neuron clusters showed high relative angiogenesis related genes in comparison to *Ntrk1*-non-expressing cell clusters (Fig. 5d).

Having established a set of secreted pro-angiogenic factors expressed in situ within mouse sensory neurons, we next examined the extent to which these factors would be over-expressed by innervating sensory nerves after HO induction (Fig. 5e–h). Here, ipsilateral lumbar DRGs were harvested 3 days after HO induction in comparison to uninjured control animals (Fig. 5e). Analysis of 15 pro-angiogenic genes by qRT-PCR showed dynamic modulation of most candidates, with significant change in 7 of 15 genes (Fig. 5f). Those genes with the highest increase in relative expression after HO induction included *Fgf18*, *Pdgfc, Vegfa*, and *Vegfd* (Fig. 5g). To confirm these transcriptional changes, immunofluorescent staining of these factors was pursued among innervating DRGs from the ipsilateral lumbar levels (Fig. 5h). Results confirmed a relative increase in immunostaining for all four pro-angiogenic proteins among HO site innervating DRGs (Fig. 5h).

### HO induction results in loss of an anti-angiogenic environment

**Fig. 6:**
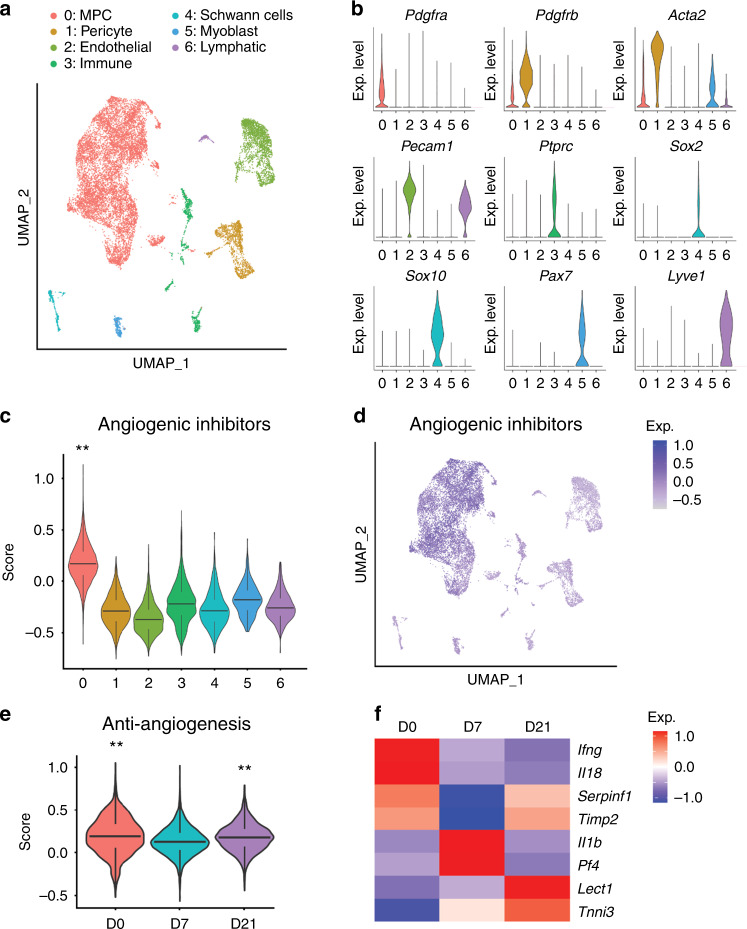
HO induction prohibits anti-angiogenesis phenotype. Single cell RNA sequencing analysis of pooled cells from the injury site, obtained at 0, 7 and 21 days post-injury. **a** UMAP visualization of seven cell clusters including mesenchymal progenitor cells (MPCs), pericytes, endothelial cells, pericytes, immune cells, Schwann cells, myoblast and lymphatic cells. **b** Violin plots demonstrating characteristic markers of each cell cluster. *Pdgfra* (Platelet-derived growth factor receptor alpha), *Pdgfrb* (Platelet-derived growth factor receptor beta), *Acta2* (Actin alpha 2), *Pecam1* (Platelet endothelial cell adhesion molecule 1), *Ptprc* (Protein tyrosine phosphatase receptor type c), *Sox2* (SRY-box transcription factor 2), *Sox10* (SRY-box transcription factor 10), *Pax7* (Paired box 7), *Lyve1* (Lymphatic vessel endothelial hyaluronan receptor 1). **c** Module score analysis of angiogenic inhibition across seven cell clusters. Gene lists for modular scores are shown in File S1. **d** UMAP visualization of expression of anti-angiogenic factors across 7 cell clusters. **e** Module score analysis of angiogenic inhibition across time. **f** Heatmap of 8 anti-angiogenic genes across time, including *Ifng* (Interferon gamma), *Il18* (Interleukin 18), *Serpinf1* (Serpin family F member 1), *Timp2* (Timp metallopeptidase inhibitor 2), *Il1b* (Interleukin 1 beta), *Pf4* (Platelet factor 4), *Lect1* (Chondromodulin), and *Tnni3* (Troponin I3). ** *P* < 0.01, non-parametric data was analyzed using the Kruskal–wallis with Dunn’s multiple comparison test (**c**, **e**)

In contrast to the highly vascular environment of heterotopic bone, tendons are, for the most part, a non-vascularized tissue. Maintaining this state under homeostatic conditions may rely on the release of soluble factors by local cells. To better understand the potential balance between neural-derived angiogenic factors and potential anti-angiogenic factors present within the local tissues, we returned to our scRNA-Seq dataset of the HO induction site, analyzed at 0, 7 and 21 days after injury (Fig. 6). Seven basic cell clusters were identified by characteristic gene markers, including mesenchymal progenitor cells (MPCs), endothelial cells, pericytes, Schwann cells, immune cells, skeletal muscle cells and lymphatic endothelium (Fig. 6a, b). A literature review identified a number of secreted molecules related to angiogenic inhibition, (including *Lect1, Ifng, Il18, Il1b, Serpinf1, Pf4, Timp2 and Tnni3*),^38^ which identified overall transcriptional enrichment in MPCs, as visualized by violin plot and UMAP projection (Fig. 6c, d). Other well characterized anti-angiogenic factors showed little or no expression in our dataset, including *Plg*, and *Il12*. Having demonstrated that tendon-associated MPCs were most enriched for anti-angiogenic factors in a quiescent state, we next examined changes in factor expression across time after injury (Fig. 6e, f). Further analysis of the MPC cluster showed that overall anti-angiogenic molecule expression was significantly reduced after injury at day 7 and starting to return to basal levels at day 21 (Fig. 6e). Of the 8 genes analyzed, *Ifng* and *Il18* were the most upregulated among MPCs within the uninjured tissues, whereas *Serpinf1* and *Timp2* have higher expression at both baseline and day 21. Lastly, *Lect1* and *Tnni3* were upregulated at day 21 which may indicate a specific role in the resolution of healing and tendon re-growth (Fig. 6f). These transcriptional results preliminary suggest a balance of angiogenic and anti-angiogenic factors which collectively may play a permissive role in vascular invasion at sites of trauma—what appears to be a necessary feature for HO formation.

## Discussion

Crosstalk between nerves and blood vessels is a key process during tissue development and repair,^8^ and based on our studies neuron-to-vessel signaling appears to be a crucial cellular event lying upstream of the differentiation of MPCs into chondrocyte-like cells and eventually into bone tissue.^4,5^ Despite this observed phenomenon in several contexts and suggestive motifs, the distinct mechanisms delineating the signaling between nerves and blood vessels in acute extremity polytrauma has remained incompletely characterized. Here, we demonstrated through a series of analysis in scRNA sequencing datasets and animal models of HO with or without inhibition of nerve signaling, that loss of neural input shifts endothelial cells toward a less proliferative and less angiogenic phenotype. In this context, the main signaling pathways that are affected by nerves in endothelial cells appear to include FGF, WNT and VEGF signaling. In addition, we observed that innervating peripheral afferent neurons upregulate angiogenic factors upon trauma, and identified several candidate growth and differentiation factors of the VEGF, PDGF and FGF signaling pathway that may play a role in neuron-to-vascular crosstalk after extremity injury.

Although four candidate factors that mediate nerve-to-vessel signaling after extremity trauma were identified, it is important to recognize that all paracrine mediators also have known and direct roles in the regulation of bone formation. For example, FGF18 is crucial for long bone regeneration. Impaired FGF18 signaling reduced bone regeneration and showed a decreased in RUNX2 and OCN expression, in this case without effect on angiogenesis.^39^ Similarly, VEGFA and VEGFD^40^ have been well studied in the roles in the regulation of endochondral ossification^29,41–43^ and osteoblastogenesis.^44^ Lastly, PDGFC is the ligand of PDGFRα which is a key regulator of osteogenesis and plays a role in endochondral ossification.^45^ Indeed, studies of *PDGFC* knockout mice showed an embryonic lethal phenotype likely by dysregulation of craniofacial structures and internal organs.^46^ In this manner, it is likely that peripheral afferent neurons have dual regulatory effects both on mesenchymal/osteoblastic and endothelial populations after trauma, coordinately allowing for a permissive environment for HO. It is worth noting that our model only entails experimental disruption of nerves and the consequent changes to vasculature. Future studies must examine the inverse vessel-to-nerve relationship, and whether vasculogenesis and peripheral neurogenesis after extremity trauma are truly interdependent processes.

Although the present work focused on only two cell types, we recognize that there are numerous additional cellular contributors to the HO niche that were not considered here. Most obviously, the interaction of immune cells in our model was not specifically assessed. The inflammatory environment leading to HO is correlated to increased levels of the inflammatory cytokines TNFα, IL-1β, IL-6, and MCP-1.^47^ Various studies have shown that immune cells including macrophages, T lymphocytes and B cells play a role in the development of HO and that their depletion reduces ossification.^48–50^ The dependence of HO on myeloid cells is well documented, including myeloid derived TGFβ.^51,52^ Along these lines, other investigators have observed monocyte/macrophage infiltration of peripheral nerves in the context of BMP2 induced heterotopic bone.^51,53^ In our prior observations, neurectomy led to subtle shifts in myeloid and populations by scRNA-Seq,^6^ and the extent to which these immune cell alterations in number, phenotype or activity would contribute to tissue repair as opposed to HO remain unclear. Additionally, mesenchymal progenitor cells themselves are important paracrine mediators of HO which release factors, such as TGFβ and BMPs, which promote osteochondral differentiation.^54^ Likewise, bone marrow is an important cellular element of maturing HO, and it is as yet unclear how sensory nerves may regulate this tissue type within HO.

Finally, our scRNA-Seq analysis implicates both pro- and anti-angiogenic factors that may coordinately control vascular ingrowth after HO induction. Of these factors, *Il18* demonstrated highest levels among uninjured tendon-associated MPCs, and whose expression was lost after HO induction. Across models, IL-18 has been shown to be an angiogenesis inhibitor,^55,56^ as well as play a role in the promotion of osteoclast formation.^57^ On the other hand, *Lect1*, a known inhibitor of angiogenesis in cartilage tissue, was upregulated at day 21 post-injury. *Lect1* is expressed in chondrocytes and promotes endochondral ossification by regulating angiogenesis.^58–61^ Together, these results suggests the local production of anti-angiogenic molecules such as *Il18* and *Lect1* in playing a role in controlling the angiogenic response to injury—potentially leading to a permissive environment for HO genesis or disease progression. Importantly, these data were obtained via transcriptomic analysis only, and future studies will need to parse out the various contributions of pro- and anti-angiogenic factors in the context of HO formation. Nevertheless, this work provides a blueprint for possible molecular targets involved in the regulation of angiogenesis during HO formation.

In summary, we provide a novel mechanism for neuro-angio crosstalk in the context of extremity polytrauma and injury at risk for HO formation, and expands our understanding on how this process is regulated from nerves to blood vessels. Importantly, it is not clear at present if a threshold for innervation levels is present for HO formation (such as is the case for BMP signaling in HO^62^), or contrarily if a more linear relationship exists between degree of innervation and amount of HO. Nevertheless, given the similarities in models of neuroskeletal communication that our group has explored previously,^4,5,63^ it is entirely possible that similar systems govern skeletal tissue morphogenesis and repair. With a greater understanding of these intertwined regulatory pathways and new molecular targets, we can optimize therapeutic strategies in the mitigation of human post-traumatic HO. More studies are needed to understand the effect of nerves and blood vessels on ossification and whether the specific inhibition of neural signaling may prevent this pathology without a delay in wound healing.

## Materials and methods

### Animal use

All animal procedures were carried out in accordance with the guidelines provided in the Guide for the Use and Care of Laboratory Animals from the Institute for Laboratory Animal Research (ILAR, 2011) and were approved by the Institutional Animal Care and Use Committee (IACUC) of the University of Texas Southwestern Medical Center (2020–102991) or Johns Hopkins University (MO19M366). All animals were housed in IACUC-supervised facilities at 18 °C–22 °C, 12 h light-dark cycle with ad libitum access to food and water, unless otherwise stated. Wild-type C57BL/6 J mice were purchased from Jackson Laboratories (Bar Harbor, ME). TrkA^F592A^ mice were donated from the Ginty laboratory, which are homozygous for a phenylalanine-to-alanine point mutation in exon 12 of the mouse *Ntrk1* gene (F592A).^64^ This point mutation in TrkA^F592A^ mice renders the endogenous TrkA kinase sensitive to inhibition by the membrane-permeable small molecule 1NMPP1.^64^ Male and female, 6–8 week old animals were used for all experiments. Wherever feasible, littermate analysis was performed while blinded to genotype.

### Surgical procedures

Trauma induced HO was achieved via complete transection of the Achilles tendon with concomitant 30% TBSA (total body surface area) partial thickness burn of the dorsum, which induces endochondral ossification of the tenotomy site over a 9-week period, as per our prior reports.^31–33^ In select experiments, surgical denervation was performed at the same time as HO induction. Proximal sciatic neurectomy was performed via a lateral thigh incision. Blunt dissection was performed through the biceps femoris to expose the sciatic nerve. The sciatic nerve was transected with reflection and suturing of the proximal nerve stump to the nearby semitendinosus muscle to prevent re-innervation. As a control, sham nerve injuries consisted of surgical exposure of the sciatic nerve without transection. Incisions were sutured closed with 5–0 Vicryl suture.

In order to achieve temporal inhibition of TrkA catalytic activity in TrkA^F592A^ animals, the small molecule 1NMPP1 was used^4–6,65^ (Aurora Analytics, LLC, Baltimore, MD). Purity (99.2%) was confirmed by HPLC-UV254, and characterization by ^1^H NMR (400 MHz, DMSO-d6) was consistent with structure. Stock solution was prepared at 200 mmol·L^−1^ by dissolving 1NMPP1 in dimethyl sulfoxide (DMSO). For 1NMPP1 administration, intraperitoneal (IP) injections were performed 24 h before, 2 h before and 24 h after injury using a 5 mmol·L^−1^ solution at a dosage of 17 μg per g body weight. In all cases, DMSO containing vehicle was used for control treatment. Animals were thereafter maintained on 1NMPP1 containing drinking water (40 μmol·L^−1^ 1NMPP1 in ddH_2_O with 1% PBS-Tween 20).

In order to achieve systemic inhibition of TrkA in C57BL/6 J animals, the TrkA inhibitor AR786 was used^6,66,67^ (Array Biopharma, Boulder, Colorado, USA). Stock solution was prepared at 750 mg·mL^−1^ of AR786 in DMSO and resuspended in corn oil to make a mixture of 2% DMSO/Corn oil. For AR786 administration, oral gavage was performed and mice were given 100 µL of the mixture at a dosage of 60 mg·kg^−1^ daily for 3 weeks. In all cases, 100 µL of 2% DMSO/Corn oil was used for vehicle control treatment.

### RNASeq—10× Single cell genomics

Three scRNA-Seq re-analyses were performed. Firstly, changes in vascular cell expression after HO induction were analyzed using a previously published dataset from our group (Accession number: GSE126060),^51^ in which the tendon injury site is examined at day 0, 7 and 21 after injury. *n* = 3 mice per timepoint were used (6–10 weeks male C57BL/6 J mice), a total of 10 119 cells, 3 815 from day 0, 3 144 from day 7 and 3 160 from day 21 total cells were examined. Downstream analysis steps were performed using Seurat as previously described.^29^ Briefly, cells with fewer than 500 genes, with more than 60 000 UMIs, or expressing a fraction of mitochondrial UMIs higher than 0.1, were filtered for quality control. Genes present in less than 3 cells per set were discarded. Unsupervised clustering (Louvain algorithm) was used to identify cell populations. Vascular clusters were identified by the expression of known marker of endothelium (*Pecam1, Chd5, Cd34*) and pericytes (*Pdgfrb, Acta2, Mcam*). A total of 4 237 vascular cells was examined. Clusters were subsequently classified by time point to characterize changes in gene expression over time. For pathway analyses, KEGG pathways and manual annotation was used to generate gene lists of pathway activators, and Metascape was used for enrichment analysis. Module scoring was based on manually curated list of genes of specific pathways or biological process, with module lists shown in File S1.

Secondly, vascular cells were examined after HO induction with or without concomitant sciatic nerve transection or sham surgery using a previously published dataset from our group (Accession number: GSE163446).^6^ *n* = 3 mice per group (6–8 weeks male and female C57BL6/J mice), 8 042 and 8 781 total cells for sham and neurectomy, respectively, obtained 7 d post-injury). As above, cells with fewer than 500 genes, with more than 60 000 UMIs, or expressing a fraction of mitochondrial UMIs higher than 0.1, were filtered for quality control. Genes present in <3 cells per set were discarded. Unsupervised clustering (Louvain algorithm) was used to identify cell populations. Cell type classification for every cluster was performed by expression analysis of canonical markers of pericytes (*Mcam, Pdgfrb, Acta2*) and endothelial cells (*Pecam1, Cdh5, Cd34*). All non-vascular clusters were removed from the dataset. Clusters were then classified and compared based on sham and neurectomy.

Finally, mouse dorsal root glial cells (DRGs) single nuclei RNA sequencing (snRNASeq) dataset was obtained from the NCBI database (accession number: GSE154659).^37^ *n* = 5 mice (8–12 weeks old male C57BL/6 J mice), 11 191 neuronal DRG cells were examined. Data were analyzed as described above. Briefly, cells with fewer than 500 genes, with more than 60 000 UMIs, or expressing a fraction of mitochondrial UMIs higher than 0.1, were filtered for quality control. Genes present in <3 cells per set were discarded. Sensory nerves subsets were identified as Neurofilament (NF, *Pvalb, Nefh*), peptidergic nociceptors (PEP, *Calca, Tac1, Ntkr1*), non-peptidergic (NP, *Mrgprd*, *P2rx3*) and tyrosine hydroxylase (TH, *Th*). After filtering every cluster, the following number of cells were analyzed for angiogenic gene expression: NF: 4 276, PEP: 4 159, NP: 4 144, and TH: 2 094. Downstream analysis steps were performed using Seurat.

### Real-time polymerase chain reaction

RNA was isolated from microdissected ipsilateral lumbar DRGs (L4-L6) from mice with or without HO induction using the GenElute^™^ Single Cell RNA Purification Kit (Sigma). Next, RNA was reverse transcribed into cDNA by iScript cDNA Synthesis Kit (Bio-Rad) following manufacturer’s instructions. SYBR Green PCR Master Mix (Life Technologies) was used for quantitative RT-PCR. See Table S1 for primer information. *n* = 3 animals per group were used, with all studies performed in biologic triplicates.

### Histology and immunohistochemistry

Hind limbs were harvested and placed in 4% paraformaldehyde (PFA) at 4 °C for 24 h. After three sequential washes in PBS, samples were decalcified in 14% EDTA (1:20 volume, Sigma-Aldrich) for 14–28 d at 4 °C. Sagittal sections of the Achilles tendon were obtained using cryosections at 10 or 50 µm thickness. Thick sections (50 µm) were used for immunohistochemical stains of nerve fibers and blood vessels. For cryosections, samples were cryoprotected in 30% sucrose overnight at 4 °C before embedding in OCT (Tissue-Tek 4583, Torrance, CA). Sagittal sections were mounted on adhesive slides (Superfrost^™^, Fisherbrand, Pittsburgh, PA). For immunohistochemistry, sections were washed in PBS × 3 for 10 min. Then sections were permeabilized with 0.5% Triton X-100 for 30 min. Next, 5% normal goat serum was applied for 30 min, then incubated in primary antibodies overnight at 4 °C in a humidified chamber (see Table S2). The following day, slides were washed in PBS, incubated in the appropriate secondary antibody for 1 h at 25 °C, then mounted with DAPI mounting solution (Vectashield H-1500, Vector Laboratories, Burlingame, CA).

Lumbar DRGs (L4-L6) were isolated from the ipsilateral side of HO induction at 3 d post-injury. The DRGs were fixed in 4% PFA overnight, washed in PBS. The DRGs were then cryoprotected in 30% sucrose overnight at 4 °C before embedding in OCT (Tissue-Tek 4583, Torrance, CA). 20 µm thick sections were used for the immunohistochemical stains. Digital images of these sections were captured with 20–100 × objectives using confocal microscopy (Zeiss LSM780 FCS or LSM800 GaAsP Carl Zeiss Microscopy GmbH, Jena, Germany).

### Histologic image analysis and histomorphometry

Images for quantification were obtained with confocal microscopy (Zeiss LSM780 FCS, or LSM800 GaAsP, Carl Zeiss Microscopy GmbH). TUBB3^+^ nerve fibers, CD31^+^, Emcn^+^ and CD146^+^ blood vessels were quantified using three-dimensional volumetric analysis of Imaris software v9.3 (Oxford Instruments, Belfast, UK) using eight serial fields per sample within the injured tissue, which included the distal tenotomy site and immediately surrounding tissues. For quantification of angiogenic factors within the DRG, cross sections of the whole ganglia were used with Leica DM6 B (Wetzlar, Germany) imaging and ImageJ software.

### Statistics

Quantitative data are expressed at mean ± SD, with *P < 0.05 and **P < 0.01 considered significant. The number of samples are indicated in figure legends. A Shapiro–Wilk test for normality was performed on all datasets. Homogeneity was confirmed by a comparison of variances test. Parametric data was analyzed using an appropriate Student’s *t* test when two groups were being compared, or a one-way ANOVA was used when more than two groups were compared, followed by a post-hoc Tukey’s test to compare two groups. Non-parametric data (modules) was analyzed using the Kolmogorov–Smirnov test. Sample size calculations were performed for experiments presented in Figs. 1, 3, 4 were based on an anticipated effect size range of 2.66–3.75, using our previously published data in adult mice.^4,6^ For this scenario, with three samples per group, a two-sample *t*-test would provide 80% power to detect effect sizes of at least 2.0 assuming a two-sided 0.05 level of significance. All sample size calculation was performed by using G*Power version 3.1.9.2 (Franz Faul, Universitat Kiel, Germany).

## Supplementary information

Supplementary Figures and Tables

Supplementary Data
