# Supplementary material for: Neuron-to-vessel signaling is a required feature of aberrant stem cell commitment after soft tissue trauma
Source: Bone Res. 2022 Jun 1;10:43. doi: 10.1038/s41413-022-00216-x (PMC9156761; doi:10.1038/s41413-022-00216-x)
Supplement: Supplementary file 1 — Supplementary Figures and Tables [file 41413_2022_216_MOESM1_ESM.docx]

**Supplementary Figure S1:**

**
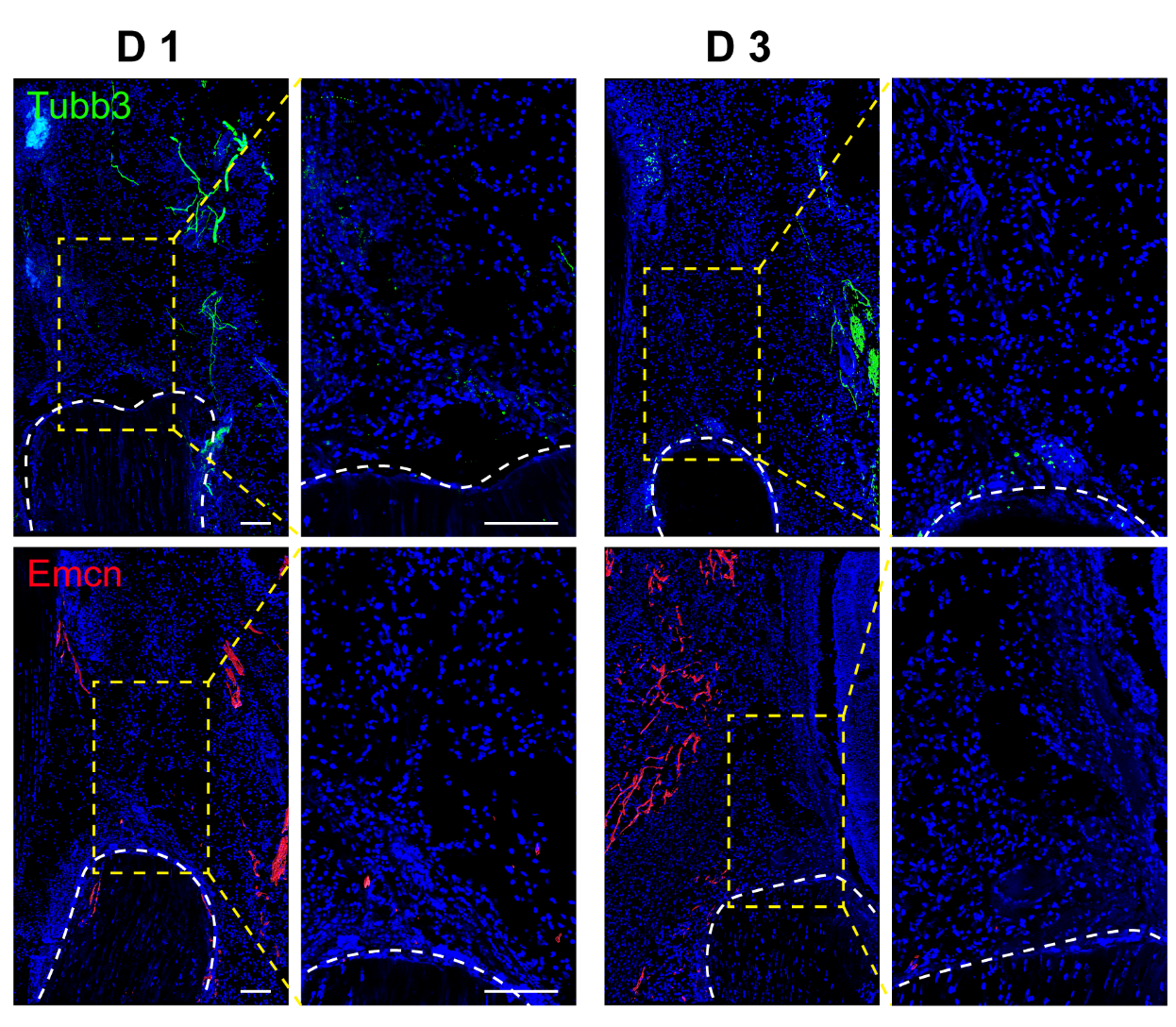
**

**Supplementary Fig. 1. Tubb3 and Emcn immunostaining** inset **following injury site at D1 and D3.** White dished line: tendon. Yellow box: high magnification inset. Scale bar: 100 μm.

**Supplementary Figure S2:**


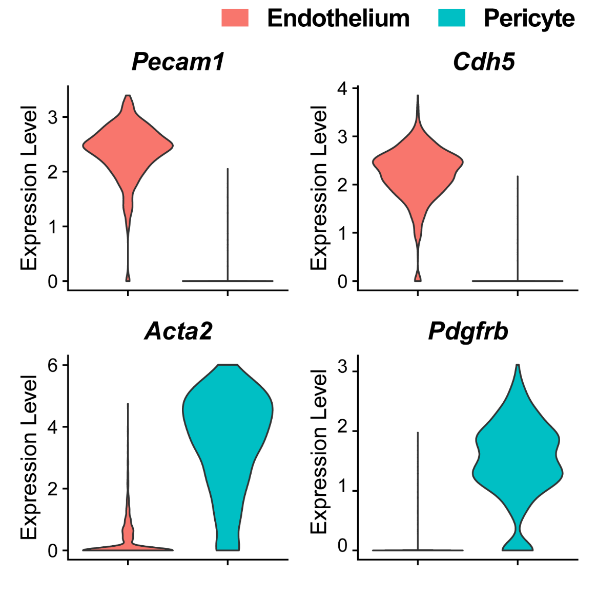


**Supplementary Fig. 2. Violin plots of characteristic markers identifying endothelial cells and pericytes within the HO induction site.** Shown in relation to data in **Figure 1E**. Cells pooled from the tendon injury site at D0, 7 and 21. N=3 animals per timepoint. *Pecam1* (Platelet endothelial cell adhesion molecule 1), *Cdh5* (Cadherin 5), *Acta2* (Actin alpha 2), *Pdgfrb* (Platelet-derived growth factor receptor beta).

**Supplementary Table S1: Primers used**

| **Genes** | **Forward (5’-3’)** | **Reverse (5’-3’)** |
| --- | --- | --- |
| *Anxa3* | ATGGCCTCTATCTGGGTTGGA | CAAGTCCTCTGATCGCTTTCC |
| *Fgf1* | TTATACGGCTCGCAGACACC | TCTGGCCATAGTGAGTCCGA |
| *Fgf18* | CTGCGCTTGTACCAGCTCTAT | GACTCCCGAAGGTATCTGTCT |
| *Mmp9* | GGACCCGAAGCGGACATTG | CGTCGTCGAAATGGGCATCT |
| *Pdgfa* | TGGCTCGAAGTCAGATCCACA | TTCTCGGGCACATGGTTAATG |
| *Pdgfb* | CATCCGCTCCTTTGATGATCTT | GTGCTCGGGTCATGTTCAAGT |
| *Pdgfc* | GCCAAAGAACGGGGACTCG | AGTGACAACTCTCTCATGCCG |
| *Pdgfd* | ATGCAACGGCTCGTTTTAGTC | CGGAGTCGCAAAAGTGTCC |
| *Pgf* | AGTGGAAGTGGTGCCTTTCAA | GTGAGACACCTCATCAGGGTA |
| *Sema5a* | GACTTGCTAGGCCCGAGAC | TCTGAACTCCCGTAACCAGGG |
| *Vegfa* | CGGGCCTCGGTTCCA | GCAGCCTGGGACCACTTG |
| *Vegfb* | GCCAGACAGGGTTGCCATAC | GGAGTGGGATGGATGATGTCAG |
| *Vegfc* | GTGAGGTGTGTATAGATGTGGGG | ACGTCTTGCTGAGGTAACCTG |
| *Vegfd* | CCTGGGACAGAAGACCACTC | TGAGATCTCCCGGACATGGT |
| *Wnt5a* | CAACTGGCAGGACTTTCTCAA | CCTTCTCCAATGTACTGCATGTG |
| *Gapdh* | CTGGGCTACACTGAGCACC | AAGTGGTCGTTGAGGGCAATG |

**Supplementary Table S2: Antibodies used.**

| **Antibody** | **Company** | **Catalog #** |
| --- | --- | --- |
| Rabbit anti-CD146 | Abcam | ab75769 |
| Rabbit anti-CD31 | Abcam | ab28364 |
| Rat anti-EMCN | Santa Cruz | sc-65495 |
| Rabbit anti-FGF18 | Thermo Fisher | 11495-1-AP |
| Mouse anti-PDGFC | R&D Systems | AF1447 |
| Rabbit anti-TUBB3 | Abcam | ab18207 |
| Mouse anti-VEGFA | Abcam | ab1316 |
| Rabbit anti-VEGFD | Abcam | ab155288 |
| Goat anti-Mouse AF488 | Abcam | ab150117 |
| Goat anti-Rabbit AF488 | Abcam | ab150077 |
| Goat anti-Rabbit DyLight 594 | Vector Laboratories | DI-1594 |
| Goat anti-Mouse AF647 | Abcam | ab150119 |
| Goat anti-Rabbit AF647 | Abcam | ab150079 |
| Goat anti-Rat AF594 | Abcam | ab150160 |
